# Propeller Flaps for Acute Lower Limb Reconstruction After Trauma: Evidence from a Systematic Review

**DOI:** 10.3390/jcm14176288

**Published:** 2025-09-05

**Authors:** Sara Matarazzo, Beatrice Corsini, Silvia Cozzi, Annachiara Tellarini, Luigi Valdatta, Ferruccio Paganini

**Affiliations:** Division of Plastic and Reconstructive Surgery, Department of Biotechnology and Life Sciences, University of Insubria, 21100 Varese, Italy; smsaramatarazzo@gmail.com (S.M.); silvia.cozzi.94@gmail.com (S.C.);

**Keywords:** propeller flap, lower limb reconstruction, trauma, perforator flap, acute wound coverage, venous congestion, flap survival, soft tissue reconstruction

## Abstract

**Background:** Propeller perforator flaps (PPFs) have gained increasing popularity in lower limb reconstruction. While their use in elective settings is well described, their role in acute post-traumatic reconstruction remains less defined. **Methods:** A systematic review was conducted following PRISMA 2020 guidelines. PubMed, Scopus, and Cochrane Library were searched on 2 June 2025, for studies reporting on the use of propeller flaps in lower limb reconstruction after trauma. Only studies rated as “good” quality using the NIH quality assessment tool were included. Data on anatomical location, flap survival, complications, reinterventions, and functional and patient-reported outcomes were extracted and analyzed descriptively. **Results:** Twenty-eight studies published between 2008 and 2024 were included, accounting for 619 propeller flaps in a population of 838 patients. The majority of flaps were fasciocutaneous, with the posterior tibial artery being the most commonly used source vessel. Among the flaps included, 422 (68.2%) achieved complete survival without necrosis, 84 (13.6%) developed partial necrosis, and 23 (3.7%) failed completely. Considering all flaps that remained viable after any required revisions or conservative management, the overall survival rate was 97%. Venous congestion was the leading cause of flap compromise. The overall complication rate was 21.8%, increasing to 35.1% in acute trauma cases. A statistically significant correlation was found between wide rotation angles (≥150°) and higher complication rates (*p* = 0.015). The mean follow-up duration was 12.5 months. Functional and aesthetic outcomes were poorly reported, but when available, they were generally favorable. **Conclusions:** PPFs represent a valuable option for lower limb reconstruction, providing reliable coverage while preserving major vascular axes. Their application in acute trauma settings appears promising, although current evidence is limited by small verified cohorts and predominantly retrospective study designs. Despite higher complication rates in acute cases, flap survival remains consistently high, supporting their use in carefully selected patients. Further prospective studies with standardized outcome reporting are needed to clarify long-term functional results and refine selection strategies.

## 1. Introduction

The reconstruction of soft tissue defects in the lower limb, particularly following trauma, represents a major challenge in reconstructive surgery [1,2,3]. Complex wounds often present with exposed bone, tendons, or implants, and require well-vascularized coverage to promote healing, prevent infection, and preserve limb function [4]. In this context, the choice of the most appropriate reconstructive option must take into account not only the defect’s characteristics but also the patient’s comorbidities, injury mechanism, available surgical resources, and timing of reconstruction [5,6].

Over the past two decades, perforator-based propeller flaps (PPFs) have emerged as a reliable and versatile tool in lower limb reconstruction [7,8,9,10,11]. First introduced by Hyakusoku et al. in 1991 [12,13] and later popularized through the work of Pignatti and colleagues [14], propeller flaps are designed around a single perforating vessel and rotated up to 180° to cover adjacent defects. Their principal advantages include preservation of major vascular axes, minimal donor site morbidity, and the possibility of harvesting local tissue without the need for microsurgical anastomosis [7,15]. The standardization of terminology and technique was advanced in the “Tokyo Consensus” [14], defining a propeller flap as an *island flap that reaches the recipient site through axial rotation* and classifying them by vascular pedicle type, rotation angle, and artery of origin [16].

These features make propeller flaps particularly attractive in the acute post-traumatic setting, where patients often present with unstable hemodynamics, inflamed or contaminated wounds, and where time- or resource-limited environments may preclude the use of free flaps [16,17]. Despite their technical simplicity, however, propeller flaps are not exempt from complications—particularly venous congestion and partial necrosis, which can compromise outcomes if not properly managed [18,19]. While the literature on PPFs is rapidly expanding, many studies include mixed indications or elective cases, limiting the generalizability of their findings to acute trauma. Furthermore, variation in surgical technique, patient selection, and outcome reporting makes it difficult to draw definitive conclusions regarding their performance in emergency scenarios [20]. Focusing specifically on trauma-related reconstructions—particularly those performed in the acute phase—allows for a more precise assessment of flap reliability, complication profiles, and patient outcomes in biologically hostile, time-sensitive contexts. By applying stringent quality criteria and extracting detailed technical variables such as perforator source, rotation angle, and timing of reconstruction, this review seeks to clarify whether PPFs can represent a safe and effective alternative to free flaps in the early coverage of lower limb injuries, and to identify the patient- and flap-related factors that most influence their success and complication rates in acute trauma.

## 2. Materials and Methods

**Figure 1:**
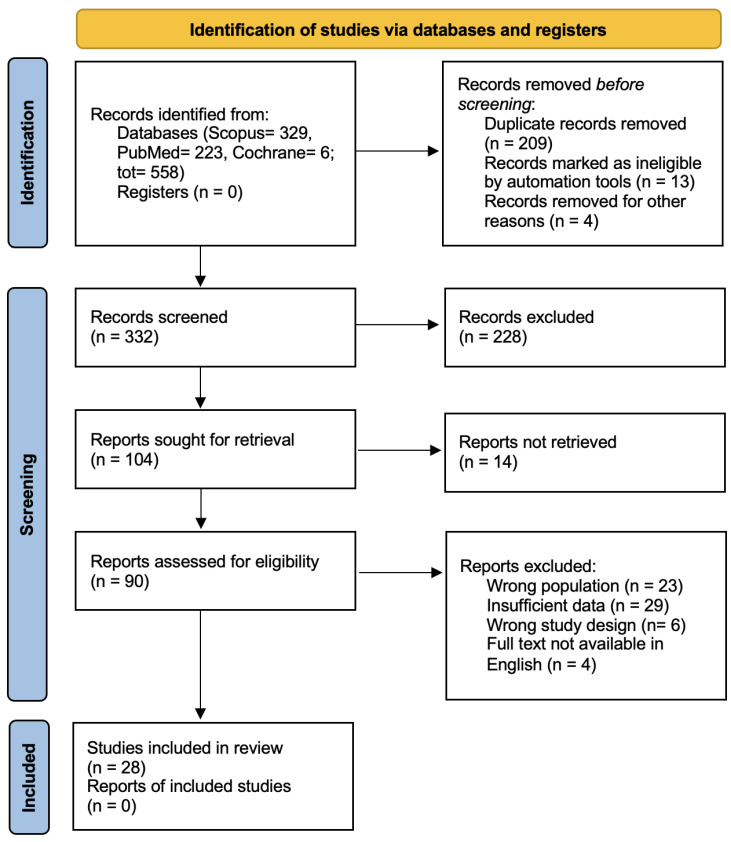
PRISMA 2020 flow diagram.

A systematic literature search was conducted on 2 June 2025, using PubMed, Scopus, and Cochrane Library databases, in accordance with the Preferred Reporting Items for Systematic Reviews and Meta-Analyses (PRISMA) 2020 guidelines [21]. The PRISMA checklist was followed and is included as Supplementary Materials. The PRISMA flow diagram is presented in Figure 1. The search aimed to identify all relevant studies published since that date. We used following search strategies for PubMed: (“*propeller*” [*title*] *OR* “*propeller flap*” [*title*] *OR* “*perforator propeller flap*” [*title*] *OR* “*pedicled perforator flap*” [*title*]) *AND* (“*trauma*” [*title*] *OR* “*traumas*” [*title*] *OR* “*traumatic*” [*title*] *OR* “*injury*” [*title*] *OR* “*soft tissue reconstruction*” [*title*] *OR* “*reconstruction*” [*title*] *OR* “*reconstructions*” [*title*]). For Scopus, we used *TITLE* (“*propeller*” *OR* “*propeller flap*” *OR* “*perforator propeller flap*” *OR* “*pedicled perforator flap*”) *AND TITLE* (“*trauma*” *OR* “*traumas*” *OR* “*traumatic*” *OR* “*injury*” *OR* “*soft tissue reconstruction*” *OR* “*reconstruction*” *OR* “*reconstructions*”), and we used (“*propeller flap*” *OR* “*perforator propeller flap*” *OR* “*pedicled perforator flap*”) *AND* (“*trauma*” *OR* “*traumatic*” *OR* “*injury*” *OR* “*soft tissue reconstruction*” *OR* “*reconstruction*”) for Cochrane.

Studies were included if they met all of the following:

- Original clinical studies (prospective, retrospective, case series or selected case reports);
- Focused on the use of propeller flaps for soft tissue reconstruction following traumatic injuries in the lower limb;
- Human studies;
- Articles written in English;
- Available as a full text.

The following types of studies were excluded:

- Cadaveric or purely anatomical studies;
- Experimental studies on animals;
- Studies where the propeller flap was used for non-traumatic indications (e.g., oncologic, pressure ulcers, burns, chronic wounds, diabetic foot);
- Reviews, letters, commentaries, expert opinions, and conference abstracts without a full text;
- Articles that did not specify the surgical technique or used ambiguous terminology (e.g., general “local flap” without mention of propeller configuration).

Articles were independently selected by three authors and then compared.

For the purpose of this review, we defined *acute* reconstruction as flap coverage performed within 21 days from injury, in line with common definitions in reconstructive literature [22]. When the timing of surgery was not explicitly stated, we inferred acute context based on descriptors such as *post-traumatic*, *initial reconstruction*, or *intra-hospital management following recent injury*. Conversely, reconstructions performed weeks to months after injury, often in healed or granulating wounds, were classified as *delayed*. Studies mixing both were classified as *mixed* timing. For transparency, all analyses were conducted on the overall trauma cohort (including both *verified* and *inferred acute cases*), while a dedicated subgroup analysis was performed on the cases with explicitly reported acute timing (*verified acute*).

It should be noted that while perforator-based propeller flaps have gained popularity in various reconstructive contexts, several authors have suggested caution in their use for acute trauma—particularly when soft tissue conditions are unstable or vascular reliability is uncertain—favoring free flaps in such scenarios [17,23,24,25]. Our review therefore aimed to evaluate whether these concerns are reflected in real-world outcomes, particularly regarding complication rates and flap survival in acute cases.

### 2.1. Data Extraction

The data extraction process was performed using a standardized Microsoft Excel spreadsheet (Microsoft Corporation, Redmond, WA, USA; Version 2210) specifically developed for this review. Three independent reviewers screened the included articles and manually extracted the relevant information, resolving any discrepancies by consensus.

For each study, bibliographic data were recorded, including first author, year of publication, journal, and study design (retrospective, prospective, case series or case report). Data on patient demographics and the number of treated cases were collected, including the total number of patients and flaps, as well as the average patient age when available.

The surgical indication was carefully assessed to distinguish cases treated in an acute post-traumatic setting from those performed in a delayed or secondary phase. Whenever this information was not explicitly stated, classification was based on timing, context, or additional descriptions in the manuscript.

Operative details included the total number of procedures, the use of preoperative imaging—such as Doppler ultrasound, CT angiography, or unspecified vascular assessments—and length of hospital stay when reported. Studies also noted whether revision surgeries were performed.

The outcome of each flap was categorized as either successful or failed, based on whether complete survival or total flap loss was reported. In addition, a detailed assessment of flap-related complications was conducted. The following adverse events were considered: hematoma, seroma, wound dehiscence, venous congestion, infection, partial necrosis, and total necrosis. When flap necrosis occurred, the presumed etiology—arterial insufficiency or venous congestion—was documented when explicitly reported or inferable from the authors’ descriptions.

The need for surgical revision of the flap was also recorded.

Data on follow-up were extracted, including the average or median duration and, when available, specific follow-up for acutely treated patients. Functional outcomes such as time to ambulation and return to normal activities were documented, as well as patient satisfaction, if explicitly mentioned.

Finally, any relevant conclusions or remarks reported by the study authors were noted to support the qualitative synthesis. In cases where data were not clearly stated or were entirely missing, the variable was marked as not reported (NR).

### 2.2. Quality Assessment

The methodological quality of the included studies was evaluated using the National Institutes of Health (NIH) Quality Assessment Tool for Observational Cohort and Cross-Sectional Studies (National Heart, Lung, and Blood Institute, 2014) [26]. This checklist includes 14 items assessing internal validity and risk of bias, such as clarity of research question, definition of the study population, outcome measures, length of follow-up, and adjustment for confounding variables. Each study was independently assessed by three reviewers. Disagreements were resolved by consensus. Based on the checklist, studies were rated as: *good* (low risk of bias, most criteria met), *fair* (some risk of bias, several criteria partially met), *poor* (high risk of bias, many criteria not met or unclear).

To ensure a high level of methodological reliability, only studies rated as *good* were included in the final synthesis. Studies rated as *fair* or *poor* were excluded from the analysis due to a higher risk of bias and insufficient reporting quality. The NIH tool was chosen for its applicability to the non-randomized, mostly retrospective design of the studies included in this review.

### 2.3. Outcome Definitions

To reduce variability across studies, outcome measures were standardized as follows. Complete flap survival was defined as full viability of the transferred tissue without any evidence of necrosis. Partial necrosis referred to any area of non-viable tissue within the flap, regardless of size, with preserved viability of the remaining portion; this was further classified as *minor* when healing occurred with conservative management or minor revision, and *major* when additional surgical coverage was required or functional compromise ensued. Complete flap loss was defined as total necrosis of the flap requiring secondary reconstruction. Complications encompassed any postoperative adverse event directly related to the flap or donor site, including venous congestion, arterial insufficiency, hematoma, seroma, infection, wound dehiscence, or lymphedema. When studies did not explicitly provide these definitions, classification was based on the authors’ descriptions and, where ambiguous, grouped according to the most conservative interpretation.

All reported outcomes in this review are flap-weighted unless otherwise specified.

## 3. Results

A total of 558 records were identified through database searches (PubMed = 223, Scopus = 329, Cochrane = 6), while no records were retrieved from study registers. After the removal of 209 duplicate records, 13 records excluded by automation tools, and 4 records removed for other reasons, 332 records were screened by title and abstract. Of these, 228 records were excluded for not meeting the inclusion criteria. The full text of 104 articles was sought for retrieval, but 14 reports could not be retrieved despite multiple attempts. Consequently, 90 articles were assessed in full text for eligibility.

Among these, 62 articles were excluded, mainly due to

- Insufficient data (*n* = 29),
- Wrong population (*n* = 23),
- Unsuitable study design (*n* = 6),
- Full text not available in English (*n* = 4).

Ultimately, 28 studies met all inclusion criteria and were included in the final qualitative analysis. (See Figure 1).

### 3.1. Descriptive Analysis

A total of 28 studies, published between 2008 and 2024, were included in this systematic review. The majority were retrospective studies, with a few case series. Across these studies, a total of 838 patients were reported. Of these, 230 were excluded from the flap-specific analysis because they were either lost to follow-up or underwent alternative reconstruction with free flaps. The remaining 608 patients received at least one propeller flap for lower limb reconstruction, accounting for a total of 619 propeller flaps. The mean patient age was 45.9 years (estimated also from studies reporting only age ranges) (see Table 1). Across all studies, male patients were more frequently represented, accounting for approximately 61% of cases. Among the comorbidities, smoking was the most commonly reported, followed by diabetes mellitus, arterial hypertension, and peripheral vascular disease.

The vast majority of flaps were performed for traumatic soft tissue defects. However, some studies also included other etiologies such as infections, oncologic resections, postoperative complications, burns, and hardware exposure.

Among the available data, at least 407 flaps were explicitly performed for post-traumatic reconstruction. Regarding timing, 39 patients were clearly reported as having undergone acute-phase procedures, accounting for 42 propeller flaps in total. Of these, 27 flaps were performed specifically for trauma-related defects and are presented as a distinct verified acute trauma subgroup in the analysis below.

The most common anatomical sites were the lower third of the leg (*n* = 174), ankle (*n* = 80), and foot (*n* = 51). Other locations included the middle third of the leg (*n* = 46), medial and lateral malleolus (*n* = 44 and 24, respectively), knee (*n* = 12), upper third of the leg (*n* = 5), and thigh (*n* = 3).

The mean defect size was approximately 46.4 cm^2^ (range: 7.5–191.1 cm^2^), while the mean flap area was 85.6 cm^2^, with an average length of 14.7 cm. This size difference likely reflects the need for tension-free closure and the geometry of the propeller design, where part of the flap remains proximal to the pivot point.

All studies reported the use of propeller flaps, mainly fasciocutaneous, with subfascial dissection. A few authors (Pignatti 2008, Zheng 2019, Innocenti 2014) also described the use of adipocutaneous flaps with suprafascial dissection [24,25,32].

The posterior tibial artery was the most commonly used perforator source (*n* = 252), followed by the peroneal artery (*n* = 122) and anterior tibial artery (*n* = 24). Rarely mentioned were the popliteal (*n* = 14), dorsalis pedis, and profunda femoris arteries.

Regarding flap rotation, most flaps were rotated between 90° and 180°, with only one study (Innocenti, 2014) [25] describing rotations under 90°. No study reported angles exceeding 180°, likely due to the increased risk of vascular compromise.

Preoperative imaging was reported in 22 out of 28 studies, primarily using handheld Doppler, with some also employing color Doppler or CT angiography. Tourniquet use was generally assumed or reported, though not always explicitly stated. General or spinal anesthesia was used depending on the setting.

Data on operative time were sparse, ranging from 60 to 160 min, with variability related to trauma complexity and surgical team experience. Length of hospital stay ranged from 7 to 21 days, when reported.

### 3.2. Clinical Outcomes

#### 3.2.1. Flaps Survival and Failure

Among the 619 propeller flaps included, 422 (68.2%) achieved complete survival without necrosis. In addition, 84 flaps (13.6%) developed partial necrosis: 33 were classified as *major*, as they required substantial surgical revision or resulted in functional compromise, while 51 were *minor*. The latter were considered as overall flap survivals in the original studies, since they healed after conservative measures or minimal revision and did not compromise the reconstruction. Complete flap loss occurred in 23 cases (3.7%). Data on flap survival were unavailable for 141 flaps (22.8%) due to incomplete reporting in several studies. Considering all flaps that ultimately remained viable after resolution of partial necrosis—whether through conservative measures or surgical revision—the overall survival rate across the entire cohort was 97%, confirming the high reliability of propeller flaps for lower limb reconstruction.

When specified, the etiology of necrosis was most commonly attributed to venous congestion (46%), likely related to pedicle torsion during flap rotation. Arterial insufficiency was reported in approximately 14% of cases. The remaining cases lacked clear documentation of vascular compromise type. (See Table 2).

#### 3.2.2. Postoperative Complications

In addition to necrosis, other postoperative complications included 14 cases of wound dehiscence, 15 infections, and 6 hematomas. No cases of seroma or lymphedema were reported. These data suggest that, while flap failure is relatively uncommon, minor and manageable complications are not rare, even in cases of successful flap survival.

#### 3.2.3. Donor Site Management

Donor site closure was inconsistently reported across studies. Six articles did not describe donor site management, while ten studies reported a combination of primary closure and skin grafting (typically split-thickness skin grafts, STSG). In three studies, primary closure alone was used, while seven reported exclusive use of skin grafts. Notably, Chaput (2018) described one case managed by secondary intention, and Franchi (2020) reported using a second propeller flap to close the donor site of the first [39,41].

These findings underscore the importance of careful donor site planning, particularly in acute lower limb reconstruction, where soft tissue availability is limited. The frequent need for skin grafting highlights the necessity for thorough preoperative counseling and intraoperative flexibility. Despite this, donor site morbidity remained generally acceptable, especially when balanced against the reconstructive efficacy of the propeller flap.

#### 3.2.4. Reintervention

Reintervention was infrequent, and generally limited to cases of complete flap failure or extensive partial necrosis. In total flap loss, free flap reconstruction was the most commonly adopted salvage strategy. In partial necrosis, most cases were managed with debridement, skin grafting, or minor local revisions, including wound revision or escarectomy. Donor site-related complications also contributed occasionally to the need for additional procedures.

#### 3.2.5. Hospitalization

Hospitalization duration was reported inconsistently, but when available, ranged between 7 and 21 days. Variability depended on comorbidities, wound contamination, and the presence of complications. While propeller flaps may not reduce the hospital stay compared to simpler techniques, they offer the benefit of single-stage definitive coverage, which may facilitate earlier rehabilitation and reduce the total recovery burden.

#### 3.2.6. Follow-Up Duration

The average follow-up period was 12.5 months, with the shortest reported follow-up being 2 months, and the longest up to 7 years. While long-term outcomes beyond 24 months were rarely available, most studies provided sufficient data to assess flap survival and early to mid-term complications, which are most relevant in the context of acute reconstruction.

#### 3.2.7. Verified Acute Trauma Subgroup

A total of 39 patients were explicitly reported as having undergone acute-phase reconstruction, accounting for 42 propeller flaps. Among these, 27 flaps were performed for trauma-related defects and were therefore included in the verified acute trauma subgroup analysis. Across these 27 flaps, outcomes were as follows: Gatto et al. reported 5 flaps, with 4 surviving completely and 1 developing partial necrosis; Ota et al. reported 6 flaps, including 1 complete loss and 1 partial necrosis; Kang et al. reported 1 flap with uneventful survival; Zhong et al. reported 15 flaps, of which 11 survived completely, while 4 developed venous congestion—2 resolving spontaneously and 2 progressing to partial necrosis; Ademola et al. described 2 flaps with complete survival and no complications; and Chiang et al. reported 1 flap with uneventful survival. Overall, in the verified acute trauma subgroup (*n* = 27), 19 flaps (70.4%) survived completely, 6 flaps (22.2%) developed partial necrosis, and 1 flap (3.7%) failed completely. Venous congestion was the most frequent complication, affecting 4 flaps (14.8%), two of which resolved without sequelae. These correspond to an overall survival rate (including flaps with resolved partial necrosis) of 96.3%.

### 3.3. Functional Outcomes and Patient Satisfaction

Functional recovery was rarely evaluated systematically. Only a few studies (Zheng 2019, Gatto 2023, Chiang 2023) included dedicated outcome measures [32,46,47]. In those reporting, patients resumed ambulation in an average of 5 to 6 weeks, with formal rehabilitation typically starting at 2 weeks postoperatively. Standardized tools such as the AOFAS score (Zheng, 2019) ranged from 82 to 98 (mean 91), suggesting excellent ankle function [32]. Overall, most patients recovered satisfactory mobility with standard footwear. However, functional outcomes may have been influenced by associated injuries, such as fractures, and were often not clearly distinguished from flap-related limitations.

Patient satisfaction was inconsistently reported. When available, results were generally favorable. In the Wang et al. (2021) cohort, Likert scale scores of 4 or 5 were assigned by the majority of patients (*n* = 72) [45]. Other studies described satisfaction qualitatively as good or satisfactory. Only two patients were explicitly described as dissatisfied, though these cases might reflect unclear reporting (potentially not reported rather than not satisfied). Overall, the available data suggest that propeller flaps are well accepted by patients, both aesthetically and functionally, even in complex reconstructions.

### 3.4. Trends in Propeller Flap Usage

**Figure 2:**
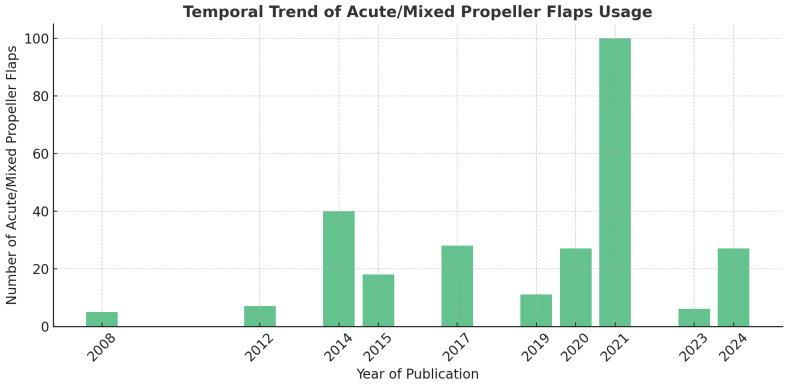
Temporal distribution of acute or mixed-timing propeller flap usage in the included studies. A progressive increase is evident from 2015 onward, with a significant peak in 2021 (100 flaps), corresponding to broader clinical adoption and multiple key publications. A renewed interest is noted in 2024 (27 flaps), reflecting increased confidence and routine use of the technique in acute trauma settings.

The analysis of publication years revealed a progressive increase in the use of propeller flaps, with a marked rise from 2015 onward (Figure 2). Between 2008 and 2014, few cases were reported, suggesting an initial experimental or limited application phase. A notable peak occurred in 2021, with 100 flaps reported—coinciding with a wider adoption of the technique and the publication of several key series. In 2024, the technique appears to have gained renewed interest, with 27 flaps published. This trend reflects growing confidence in propeller flap use, even in acute trauma settings, supported by improved surgical familiarity and evidence base.

### 3.5. Complication Rates over Time

**Figure 3:**
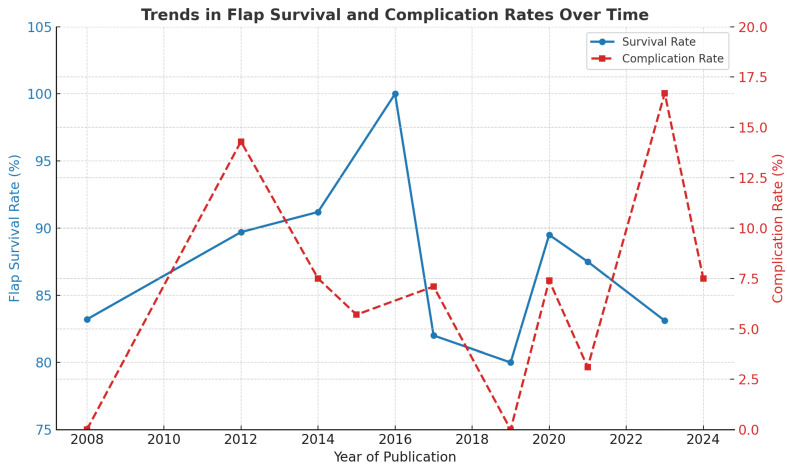
Trends in flap survival and complication rates over time in lower limb propeller flap reconstruction. The blue line indicates overall flap survival rates (%), while the red dashed line indicates overall complication rates (%). A progressive improvement in survival is observed across the years, paralleled by a gradual reduction in complications, suggesting the impact of increasing surgical experience, refined technique, and improved perioperative management.

Despite the rising number of cases, the overall complication rate remained stable or showed a mild decline, suggesting technical refinement over time (Figure 3). In 2021, the year with the most cases, the complication rate was only 3%, one of the lowest observed. Higher rates in 2012 (14.3%) and 2023 (16.7%) may be skewed by the small number of cases in those years. This inverse trend between volume and complications may reflect improved patient selection, pedicle handling, and preoperative planning.

### 3.6. Flap Survival

The overall flap survival rate (including flaps with resolved partial necrosis) across the entire cohort was 97%, confirming the high reliability of propeller flaps for lower limb reconstruction. When isolating acute trauma cases, survival was slightly lower at 96.3%, likely reflecting the more challenging context: tissue edema, vascular instability, and limited planning time. Despite this, flap success remained consistently high across settings (Figure 3).

A Cochran–Armitage test for trend revealed a statistically significant improvement in flap survival over time (χ^2^ = 44.58, *p* = 0.0000058), even though visual trends appeared stable. This statistical signal suggests that experience, improved case selection, and perioperative management may have contributed to better outcomes over the years.

### 3.7. Acute vs. Overall Complication Rate

**Figure 4:**
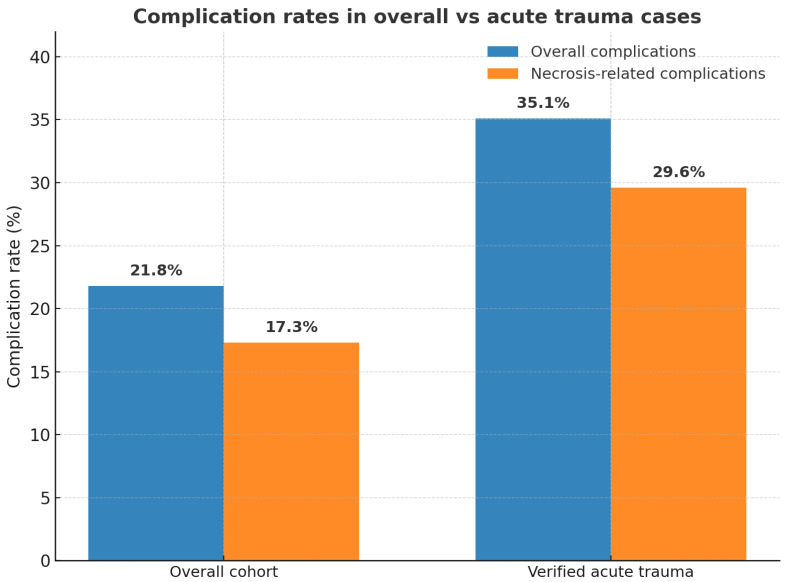
Complication rates in the overall cohort and in the verified acute trauma subgroup. Overall complication rates (blue bars) include all reported adverse events and complications while necrosis-related complication rates (orange bars) refer specifically to partial necrosis or complete flap loss. In the overall cohort, complication rates were 21.8% (overall) and 17.3% (necrosis-related). In the verified acute trauma subgroup, complication rates were higher, at 35.1% (overall) and 29.6% (necrosis-related).

The overall complication rate was 21.8%, consistent with existing literature. However, when isolating acute trauma cases, this rate increased to 35.1% (Figure 4). This value reflects the aggregate of all reported adverse events, including not only flap-related problems but also wound infections, dehiscence, and donor-site issues. When restricting the analysis to flap-specific necrosis (partial or complete, both *major* and *minor*), the complication rate in the acute trauma subgroup was 29.6%. These findings suggest that the higher morbidity observed in the acute setting is not solely attributable to flap-related complications, but also to the greater biological vulnerability of freshly traumatized wounds, with increased susceptibility to infection, inflammation, and vascular fragility.

### 3.8. Technical Factors Influencing Complications

A clear association was observed between rotation angle and complications. Flaps rotated between 90° and 120° showed no complications, while those rotated at 150° had a 66.7% complication rate, and flaps rotated 160–180° had 100% complication incidence. A chi-square test confirmed this association (χ^2^ = 12.32, *p* = 0.015), supporting the hypothesis that torsional stress on the vascular pedicle significantly increases risk.

Outcomes varied based on the perforator vessel used. Flaps based on the peroneal artery had a higher complication rate than those based on the posterior tibial artery, while anterior tibial artery-based flaps had the lowest complication rates. This variability may be due to vessel anatomy, caliber, or ease of dissection.

## 4. Discussion

This systematic review confirms that propeller perforator flaps (PPFs) are a safe, reproducible, and versatile reconstructive option for small- to medium-sized soft tissue defects of the lower limb, including in acute and emergency trauma settings [27,42,47]. Their growing use over the past decade reflects increasing surgical familiarity and expanding indications. Our temporal analysis showed a significant increase in the use of PPFs over time, with a statistically significant trend toward improved flap survival (*p* < 0.001), likely driven by improved technique, better case selection, and increased awareness of anatomical and technical nuances.

Compared to free flaps, PPFs offer significant advantages in selected cases, particularly in terms of shorter operative time, reduced donor site morbidity, and no need for microvascular anastomosis, making them especially suitable for patients with comorbidities or in resource-limited environments [31,43,48]. The present review revealed an overall flap survival rate of 97%, confirming the technique’s reliability. Even when isolating acute trauma cases, survival remained high at 93%, despite the challenging biological environment typical of emergency settings.

However, PPFs are not without limitations. The most frequently reported complications—venous congestion and partial necrosis [7,25]—are strongly associated with technical factors, such as excessive rotation angles (150°), short distance between perforator and defect (<3.5 cm) and anatomical and vascular variability (e.g., peroneal-based flaps) [45,50]. These associations were statistically confirmed (chi-square test, *p* = 0.015), reinforcing the need for conservative design and careful planning, particularly in acutely inflamed or contaminated tissues.

Importantly, we observed a higher complication rate in acute trauma cases (35.1%), reflecting the challenges of inflamed, edematous, and potentially contaminated tissue. Nonetheless, these did not translate into high failure rates, confirming the resilience and adaptability of propeller flaps when correctly planned.

Donor site closure remains a key consideration. Our findings confirm that split thickness skin graft (STSG) is frequently required, especially in larger flaps or in areas with limited skin laxity. However, overall morbidity was low, and several innovative strategies—such as the use of a second propeller flap for closure—have been described [39,41].

Importantly, functional outcomes and patient-centered metrics—such as return to ambulation, quality of life (QoL), return to work, and patient satisfaction—remain significantly underreported in the current literature. This represents a major limitation and a missed opportunity: without a clear understanding of what these reconstructions restore in terms of real-world functionality, the value of the technique remains only partially demonstrated. Future research should systematically assess these dimensions using validated tools, such as the Lower Extremity Functional Scale (LEFS) [51], EuroQol-5D (EQ-5D) [52], and work reintegration timelines. Orthoplastic success must be measured not only by flap viability but by its impact on the patient’s daily life and social reintegration.

From a technical standpoint, several refinements have been explored to increase flap reliability—such as venous supercharging [39,53], dual-perforator designs [54], flap delay [55], or preservation of surrounding adipofascial tissue [56]—with promising results in high-risk settings [28,39,45]. These should be further investigated in larger comparative studies.

Preoperative imaging deserves greater emphasis. Although color Doppler or CT angiography were rarely reported in the included studies, they may be critical in the trauma setting, where vessel trajectories are unpredictable and intraoperative dissection may be risky or time-consuming. Routine use of perforator mapping could reduce complications and optimize flap planning [57,58].

Furthermore, wide heterogeneity in flap nomenclature and classification (e.g., adipofascial vs. fasciocutaneous, axial vs. perforator-based) complicates data comparison. Standardizing these definitions and reporting parameters (rotation angle, flap length, delay technique, donor site management) is essential to strengthen the evidence base [8,59,60].

This review also highlights gaps in cost-effectiveness data. Only one study (Innocenti et al., 2019) performed a comparative economic analysis versus free flaps [40]. Given rising healthcare costs, future studies should incorporate economic endpoints, particularly when considering PPFs as alternatives to microsurgical reconstruction in trauma.

Future research should focus on prospective, multicenter studies comparing PPFs with other techniques; standardization of outcome measures, including functional and patient-reported results; the role of preoperative imaging and computational planning in improving flap safety; application of AI-based flap design algorithms and intraoperative perfusion monitoring; evaluation of technical refinements (e.g., venous supercharging, staged flap rotation, or perforator mapping protocols).

### Study Limitations

This review has several limitations. Most included studies were retrospective, with variable definitions for outcomes and inconsistent follow-up durations. The definition of acute reconstruction also varied across studies. For the purpose of this review, we considered procedures performed within 21 days from injury as acute, following precedent in the literature [17,23]. However, the true definition remains debated.

Selection bias is likely, given the lack of randomized controlled trials and the predominance of single-center experiences. Moreover, several studies lacked data on key outcomes such as operative time, comorbidities, or donor site morbidity. The absence of standardized patient-reported functional and aesthetic outcomes further limits the clinical translation of these findings. Another limitation is the absence of a pre-registered review protocol (e.g., PROSPERO), which may increase the risk of selection bias. Moreover, operational definitions for key outcomes (partial necrosis, complete loss, overall complications) varied across studies. We adopted standardized definitions for the purpose of this review and explicitly acknowledged inconsistencies as a limitation, which may affect comparability across reports.

## 5. Conclusions

This systematic review highlights the increasing relevance of perforator-based propeller flaps (PPFs) in lower limb reconstruction following trauma. Over the past decade, the technique has been increasingly adopted as an alternative to free flaps for small to moderate defects, as well as in acute settings where traditional microsurgical approaches may be limited by patient comorbidities, logistical constraints, or soft tissue conditions.

Our analysis confirms high flap survival rates, even in emergency scenarios, and demonstrates a clear trend toward improved outcomes over time—likely attributable to greater surgical experience, technical refinements, and more accurate preoperative planning. Nevertheless, complication rates—particularly in acute trauma cases—remain notable, emphasizing the importance of careful case selection, judicious flap design, and awareness of risk factors such as excessive rotation angles or perforator selection. The evidence is weakened by the fact that only a minority of studies clearly reported acute-phase reconstructions. Moreover, the retrospective design of nearly all included studies limits the ability to draw causal conclusions.

Despite these challenges, propeller flaps provide a valuable balance between surgical simplicity and reconstructive effectiveness. Their use should be encouraged in well-selected patients, with appropriate technical considerations and contingency plans. Furthermore, evolving strategies such as venous supercharging, dual-perforator flaps, and adipofascial preservation appear promising in enhancing flap safety, especially in complex or high-risk reconstructions.

Future research should aim to overcome current limitations through prospective multicenter studies, standardized outcome measures—including functional and aesthetic assessments—and the integration of modern planning tools such as advanced imaging, perfusion monitoring, and artificial intelligence algorithms. Only through these steps can the role of propeller flaps be fully optimized in contemporary trauma reconstruction.

## Figures and Tables

**Table 1:** Summary of the 28 studies included in the systematic review. The table reports key study characteristics, including study design, patient age, number of flaps, defect etiology and location, defect and flap size, timing of reconstruction, flap type, source vessels, and flap rotation angles.

Author	Study Type	Patient Age (Mean)	Number of Flaps	Etiology of Defect	Location of Defect	Size of the Defect (Longest Side; Mean Area or Range)	Timing of Reconstruction (Acute/Delayd/Mixed)	Flap Type	Source Vessel (Number and Vessel)	Flap Rotation Angle (Grades or Range)	Size of Flap (Longest Side; Mean Area or Range)
Pignatti, 2008 [24]	prospective	52.5	6	5 trauma, 1 hardware exposure	3 middle third leg, 1 lower third leg, 1 knee, 1 medial malleolus	9.8 cm; 89.83 cm^2^	mixed	FC AC	NR	90°, 135°, 180°	17.8 cm; 216.67 cm^2^
Karki, 2012 [27]	retrospective	38	20	20 trauma	10 medial malleolus, 7 lateral malleolus, 3 lower third leg	14–35 cm^2^	NR	FC	14 PTA, 6 PA	180°	NR
Mateev, 2012 [28]	case series	37	11	4 trauma, 3 burn, 2 infection, 2 tumor excision	10 lower third leg, 1 foot	NR	mixed	FC	PTA, PA, LMA, DPA	180°	52 cm^2^
Shin, 2012 [29]	retrospective	54.3	8	3 trauma, 2 ulcer, 2 tumor, 1 post-surgical	leg	5–10 cm	mixed	FC	5 PTA, 3 PA	90–180°	15cm; 135 cm^2^
Chang, 2014 [30]	case series	43	12	6 trauma, 4 infection, 1 tumor excision, 1 pressure sore	5 foot, 1 lower third leg	9–66 cm^2^	mixed	FC	4 PTA, 2 PA	180°	15.1 cm; 117.78 cm^2^
Innocenti, 2014 [25]	retrospective	54	66	27 trauma, 18 tumor excision, 17 post-surgical	knee, lower third leg, the Achilles region	NR	mixed	FC AC	PTA, ATA, PFA, PA, LCFA, MSGA, LPCA, MPA	<90°, 91–180°	10–375 cm^2^
Rogers, 2014 [31]	prospective	28.9	7	7 trauma	3 lateral malleolus, 1 medial malleolus, 2 lower third leg, 1 middle third leg	20.75 cm^2^	mixed	FC	4 PTA, 7 PA	90−180°	NR
Zheng, 2014 [32]	case series	37	5	4 trauma, 1 tumor excision	5 knee	18.2 cm; 29.76–191.1 cm^2^	NR	FC	DGA	180°	104.96 cm^2^
Ademola, 2015 [33]	case report	34	2	gunshot	leg	10 cm	acute	FC	PTA	90–180°	NR
Kang, 2015 [34]	case report	45	1	trauma	right heel	4 cm; 16 cm^2^	acute	FC	PTA	180°	NR
Zhong, 2015 [35]	case series	40	15	trauma	leg, foot	NR	acute	FC	PTA	NR	17 cm; 92.5 cm^2^
Shen, 2016 [36]	retrospective	39.7	36	trauma	12 lower third leg, 24 foot	8–120 cm^2^	NR	FC	PA	180°	50-612 cm^2^
Cajozzo, 2017 [37]	retrospective	74	17	9 trauma, 6 tumor excision, 1 infection, 1 diabetic ulcer	8 lower third leg, 6 middle third of leg, 2 popliteal fossa, 1 knee	8 cm	mixed	FC	8 PTA, 7 PA, 2 ATA	90°, 180°	24–130 cm^2^
Balakrishnan, 2017 [38]	retrospective	38	22	19 trauma, 2 hardware exposure, 1 bite	lower third leg	mainly small- and medium- sized defects	mixed	FC	PTA	180°	9.7 cm; 35.09 cm^2^
Chaput, 2018 [39]	retrospective	52.5	60	38 trauma, 14 infection, 5 tumor excision, 3 burn	13 middle third leg, 27 lower third leg, 10 foot	NR	NR	FC	33 PTA, 27 PFA	120°	48–58 cm^2^
Innocenti, 2019 [40]	retrospective	53	79	40 trauma, 21 tumor excision, 4 infection, 14 unknown	NR	NR	NR	NR	36 PTA, 11 PA, 8 PA, 8 PFA, 5 ATA, 4 MPA, 4 CFA, 3 others	NR	68 cm^2^
Zheng, 2019 [32]	prospective	34.94	18	11 trauma, 3 poor healing, 2 infection, 1 ulcer, 1 tumor excision	15 foot, 3 malleolar	NR	mixed	AC	PA	NR	14.3 cm; 53.36 cm^2^
Franchi, 2020 [41]	case series	54	16	8 trauma	4 lower third leg, 2 foot, 2 thigh	6 cm; 46.4 cm^2^	mixed	FC	7 PA, 2 PFA, 2 dbLCFA, 3 PTA, 1 ATA, 1 MSA	150–180°	12.3 cm; 73.4 cm^2^
Lese, 2020 [17]	retrospective	60	26	8 trauma, 9 post-surgical, 9 infection	lower third leg	9–36 cm^2^	mixed	NR	12 PTA, 14 PA	150–180°	68–144 cm^2^
Valente, 2020 [42]	retrospective	36.4	14	trauma	2 middle third leg, 12 lower third leg	9 cm^2^	mixed	FC	ATA, PTA	180°	29 cm^2^
Eldahshoury, 2021 [43]	retrospective	45.5	23	20 trauma, 2 infection, 1 tumor excision	20 lower third leg, 3 foot	15–154 cm^2^	NR	FC	11 PTA, 12 PA	NR	NR
Guillier, 2021 [23]	retrospective	55.4	21	18 trauma, 3 infection	2 lateral malleolus, 3 foot, 1 middle third leg, 1 medial malleolus, 3 knee, 4 upper third leg, 5 middle third leg, 2 lower third leg	29.8 cm^2^	mixed	FC	NR	90–180°	NR
Tapan, 2021 [44]	case series	37.5	11	5 trauma, 1 gunshot, 1 ulcer, 1 tumor, 1 burn, 2 post-surgical	7 ankle, 4 foot	5–10 cm	mixed	FC	PA	90–180°	16 cm; 208 cm^2^
Wang, 2021 [45]	retrospective	36.5	82	62 trauma, 20 infection	11 middle third leg, 32 lower third leg, 31 medial malleolus, 8 lateral malleolus	10.6 cm	mixed	FC	12 ATA, 62 PTA, 8 PA	<150°, 151–180°	15.6 cm; 60.7 cm^2^
Chiang, 2023 [46]	case report	20	1	trauma	leg	12 cm; 72 cm^2^	acute	FC	PTA	90–180°	12 cm; 60 cm^2^
Gatto, 2023 [47]	retrospective	33.8	5	trauma	4 lateral malleolus, 1 middle third leg	5–10 cm	acute	FC	4 ATA, 1 PA	90–170 °	12 cm
Humnekar, 2024 [48]	controlled randommize trial	31.53	17	12 trauma	7 foot, 8 lower third leg, 1 upper third leg, 1 thigh	20.60 cm^2^	mixed	FC	PA, DPA, PTA, PFA	180°	41–55 cm^2^
Ota, 2024 [49]	retrospective	58	18	6 trauma,9 post-surgical, 3 infection	3 middle third leg,13 lower third leg, 2 foot	24–80 cm^2^	acute	FC	PTA, PA	NR	NR

ATA anterior tibial artery, PTA posterior tibial artery, PA peroneal artery, DGA descending genicular artery, MSGA medial superior genicular artery, LMA lateral malleolar artery, DPA dorsalis pedis artery, PFA profunda femoris artery, MPA medial plantar artery, CFA circumflex femoral artery, descending branch of lateral circumflex femoral artery dbLCFA 2, MSA medial sural artery, LPCA lateral popliteal cutaneous artery, FC fasciocutaneous, AC adipocutaneous, NR not reported.

**Table 2:** Summary of flap survival and complications across the included studies. For each study, the number of flaps, survival rates, and specific complications (total/partial necrosis, wound dehiscence, hematoma, seroma, infection, and lymphedema) are reported.

Author	Number of Flaps	Survived Flaps	Complications (Yes/No)	Total Necrosis	Partial Necrosis	Other Complications
Pignatti, 2008 [24]	6	5	Y	0	1	0
Karki, 2012 [27]	20	19	Y	0	1	1 wound dehiscence
Mateev, 2012 [28]	11	10	Y	1	0	0
Shin, 2012 [29]	12	5	Y	0	1	0
Chang, 2014 [30]	66	65	Y	1	7	0
Innocenti, 2014 [25]	7	7	Y	0	1	0
Rogers, 2014 [31]	5	5	N	0	0	0
Zheng, 2014 [32]	2	2	Y	0	0	0
Ademola, 2015 [33]	1	1	N	0	0	0
Kang, 2015 [34]	15	11	Y	0	1	1 infection
Zhong, 2015 [35]	36	36	Y	0	9	1 hematoma, 1 infection
Shen, 2016 [36]	22	19	Y	0	0	1 hematoma
Cajozzo, 2017 [37]	17	13	Y	0	4	2 wound dehiscence, 1 infection
Balakrishnan, 2017 [38]	60	41	Y	3	10	1 infection
Chaput, 2018 [39]	79	76	Y	3	13	4 wound dehiscence
Innocenti, 2019 [40]	18	11	Y	0	1	2 wound dehiscence
Zheng, 2019 [32]	16	14	Y	0	2	1 hematoma
Franchi, 2020 [41]	26	23	Y	1	2	NR
Lese, 2020 [17]	14	12	Y	0	2	1 wound dehiscence, 2 hematoma
Valente, 2020 [42]	23	21	Y	1	1	1 wound dehiscence
Eldahshoury, 2021 [43]	21	13	Y	1	6	1 wound dehiscence, 1 hematoma
Guillier, 2021 [23]	11	9	Y	1	1	0
Tapan, 2021 [44]	8	6	Y	0	0	0
Wang, 2021 [45]	82	65	Y	6	11	11 infection
Chiang, 2023 [46]	1	1	N	0	0	0
Gatto, 2023 [47]	5	4	N	0	1	0
Humnekar, 2024 [48]	18	6	Y	2	7	NR
Ota, 2024 [49]	17	14	Y	3	3	0

NR not reported, Y yes, N no.

## Data Availability

No data collection forms, extracted data, analytic code or other materials used in this review are publicly available, but are available from the corresponding author upon reasonable request.

## Supplementary Materials

The following supporting information can be downloaded at: https://www.mdpi.com/article/10.3390/jcm14176288/s1, PRISMA 2020 Checklist.
